# Improvement of Photoresponse in Organic Phototransistors through Bulk Effect of Photoresponsive Gate Insulators

**DOI:** 10.3390/ma13071565

**Published:** 2020-03-28

**Authors:** Hea-Lim Park, Min-Hoi Kim, Hyeok Kim

**Affiliations:** 1 Department of Materials Science and Engineering, Seoul National University, Kwanak-gu, Seoul 151-600, Korea; haelim1017@snu.ac.kr; 2 Department of Creative Convergence Engineering, Hanbat National University, Yuseong-ku, Daejeon 305-719, Korea; mhkim8@hanbat.ac.kr; 3 School of Electrical and Computer Engineering, University of Seoul, 163 Seoulsiripdaero, Dongdaemun-gu, Seoul 02504, Korea

**Keywords:** organic phototransistor, photoresponsive polymer, polymer gate insulator, bulk effect, poly(4-vinylphenol)

## Abstract

In this study, we investigate the bulk effect of photoresponsive gate insulators on the photoresponse of organic phototransistors (OPTs), using OPTs with poly(4-vinylphenol) layers of two different thicknesses. For the photoresponse, the interplay between the charge accumulation (capacitance) and light-absorbance capabilities of a photoresponsive gate insulator was investigated. Although an OPT with a thicker gate insulator exhibits a lower capacitance and hence a lower accumulation capability of photogenerating charges, a thicker poly(4-vinylphenol) layer, in contrast to a thinner one, absorbs more photons to generate more electron–hole pairs, resulting in a higher photoresponse of the device. That is, in these two cases, the degree of light absorption by the photoresponsive gate insulators dominantly governed the photoresponse of the device. Our physical description of the bulk effect of photoresponsive insulators on the performance of OPTs will provide a useful guideline for designing and constructing high-performance organic-based photosensing devices and systems.

## 1. Introduction

Organic phototransistors (OPTs) have garnered significant attention as fundamental building blocks for optoelectronic systems adopting light as an information carrier. An OPT is a type of optical transducer wherein the functions of light detection, switching, and signal amplification are integrated into a single device. In addition to the intrinsic advantages of organic materials, such as low cost, mechanical flexibility, and chemical versatility, OPTs exhibit higher photosensitivity and lower noise than two-terminal organic photodiodes because the gate electrode, being the third terminal, enables charge carriers to be accumulated at the interface between the gate insulator and organic semiconductor layer, resulting in the amplification of output signals [1,2,3,4,5]. In these OPTs, the photoresponse can be simply tuned by adopting organic materials such as photochromes through photoisomerization of these molecules at different wavelengths and photoresponsive polymers [6,7,8,9].

In the field of organic field-effect transistors, gate insulators are critical to device performance because both charge accumulation and charge transport are affected significantly by the characteristics of the dielectric layer, such as the dielectric constant, layer thickness, and the functional groups in the molecular structures [10,11]. Regarding OPTs, many previous studies have focused on the effects of the interfacial properties of gate insulators on photoresponsive properties [12,13,14,15,16,17,18,19,20,21,22]. However, the bulk effect of gate insulators on the photoresponse has rarely been investigated, although for organic field-effect transistors, a few researchers have indicated that the bulk part of the gate insulator must be considered for determining their performance. For practical applications of OPTs, it is crucial to comprehensively understand the effects of gate insulators on the photoresponse of the device.

In this study, we first investigate how the thickness of photoresponsive gate insulators affects the photoresponse of OPTs. For the device fabrication, poly(4-vinylphenol) (PVP) was used as the gate insulator. It is noteworthy that PVP is regarded as a promising material among polymer gate insulators of OPTs owing to its excellent dielectric properties and photoresponsive characteristics under ultraviolet (UV) exposure [18,19]. In our devices, we discovered that the light absorbance capability of the gate insulator was critical to the photoresponse of the OPTs; therefore, the device with a thicker PVP layer exhibited higher photosensitivity (*P*) and photoresponsivity (*R*) compared with a thinner PVP layer.

## 2. Materials and Methods

**Figure 1:**
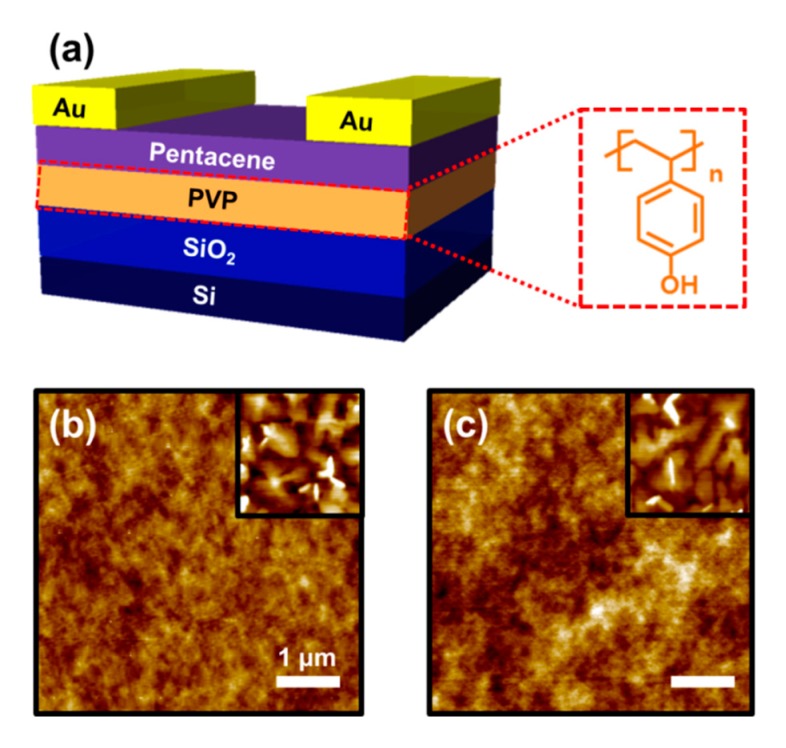
(**a**) Schematic organic phototransistor (OPT) device structure and chemical structure of poly(4-vinylphenol) (PVP). Atomic force microscopy (AFM) images of (**b**) 60 nm-thick and (**c**) 260 nm-thick PVP layers. Inset images present the AFM images of 10 nm-thick pentacene layers on each PVP layer.

The structure of the fabricated OPT is shown in Figure 1a. Thermally grown 300 nm thick SiO_2_ layers were used as first gate insulators and Si wafers as gate electrodes. In fabricating the PVP layers (the second gate insulators), PVP (*M*_w_ = 25,000 g/mol, Sigma–Aldrich, Seoul, Korea) mixed with poly(melamine-co-formaldehyde) (100 wt.% of the PVP) in propylene glycol methyl ether acetate was used. PVP solutions at concentrations of 2 and 7 wt.% were spin-coated on the SiO_2_ layers (3000 rpm, 30 s) to form the PVP layers with approximate thicknesses of 60 and 260 nm, respectively, as shown in Figure S1. The deposited PVP films were annealed at 100 °C for 30 min followed by 200 °C for 60 min in ambient conditions. On the PVP layers, pentacene (50 nm) was thermally evaporated at 0.5 Å/s and at a pressure of 1 × 10^−5^ Torr. Subsequently, Au was deposited through thermal evaporation at the rate of 1 Å/s and at a pressure of 1 × 10^−5^ Torr. During Au deposition, source and drain electrodes were defined with channel length and width of 150 μm and 1 mm, respectively, through a shadow mask. The electrical characteristics of the OPTs were measured using a semiconductor parameter analyzer (HP4155A, Hewlett–Packard Co., CA, USA) under ambient conditions. A UV light source (GL-155, UVSMT, Gyeonggi-do, Korea) with a peak wavelength of 365 nm and an intensity of 3 mW/cm^2^ was used to measure the photoresponse of the devices.

## 3. Results and Discussion

Figure 1b,c exhibit atomic force microscopy (AFM) images of 60 nm-thick and 260 nm-thick PVP layers, respectively. The root-mean-square roughness values are 0.047 for the 60 nm-thick PVP layer and 0.053 nm for the 260 nm-thick PVP layer. In the inset images of Figure 1b,c, AFM images of 10 nm-thick pentacene layers on each PVP layer are presented and they show a similar level of grain size. The reason why the 10 nm-thick pentacene film was observed for analysis of pentacene morphology is that the first few monolayers of pentacene mainly contribute to the charge transport. 

**Figure 2:**
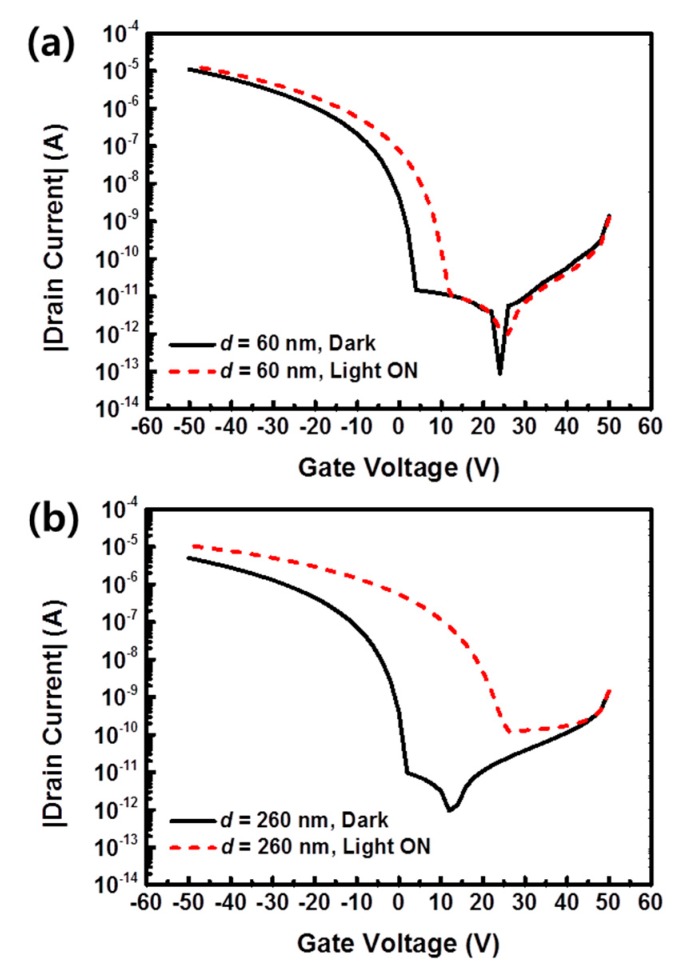
Transfer curves of the OPTs with two different thicknesses, *d*, of PVP, in the dark and under light exposure; (**a**) *d* = 60 nm and (**b**) *d* = 260 nm.

To investigate the bulk effect of the photoresponsive PVP gate insulator on the photoresponse, we fabricated two OPTs with different thicknesses (*d*) of the PVP gate insulator on the SiO_2_ insulators; the values of d in the two different devices were approximately 60 and 260 nm (see Figure 1 for the schematic structure). It is noteworthy that the SiO_2_ layers were exploited as first gate insulators such that the electrical characteristics of the OPTs were not changed significantly, even with the thinnest d of 60 nm [18,19,23,24]. As shown in Figure S2, gate leakage current levels of the two OPTs with different PVP thickness (*d* = 60 and 260 nm) were compared. No significant difference in gate leakage current between these two devices was observed. This may be attributed to the high-quality insulating property of the first gate insulator (SiO_2_) [18,19,23,24]. Figure 2 shows the transfer curves of the OPTs obtained for gate voltages (*V*_g_) from 50 to −50 V at the drain voltage (*V*_d_) of −50 V. The mobility was approximately 0.16 cm^2^/Vs for both *d* = 60 and 260 nm. The threshold voltage (*V*_th_) exhibited a more negative value of −8.4 V for the device with *d* = 260 nm, in contrast with a −7.4 V value for the *d* = 60 nm device. This was because the OPT with a thicker gate insulator (*d* = 260 nm) had a lower capacitance of 5.41 nF/cm^2^ compared with 8.08 nF/cm^2^ of the thinner device (*d* = 60 nm); therefore, a higher bias was required to turn on the device with the thicker PVP layer. For gate voltages *V*_g_ from 0 to −50 V (electrically on state), the drain current level of the OPT with *d* = 60 nm was higher than that of the *d* = 260 nm device at the same *V*_g_. This was also attributed to the higher capacitance of the gate insulator with *d* = 60 nm. On-off ratio values of *d* = 60 and 260 nm devices were 7.46 × 10^5^ and 5.20 × 10^5^, respectively. 

As shown in Figure S3, under light illumination, the mobility values were 0.18 cm^2^/Vs for the *d* = 60 nm device and 0.16 cm^2^/Vs for the *d* = 260 nm device, which showed a negligible change in both devices under light exposure. In addition, *V*_th_ values were −2.3 for the thinner device and 12.6 V for the thicker device. That is, in comparison with the *V*_th_ values before light exposure, *V*_th_ values were shifted to the positive direction with the magnitude of 5.1 V in the thinner case and 21.0 V in the thicker case. The shift of *V*_th_ to the positive direction under light illumination was reported to be proportional to the number of trapped electrons at organic semiconductor/gate insulator interfaces. As for on-off ratio, the thinner device was 1.28 × 10^6^ and the thicker device was 8.76 × 10^4^. The thicker device exhibited a highly reduced on-off ratio, and this was mainly attributed to the increased off current during light exposure from excessively photogenerated charges. 

**Figure 3:**
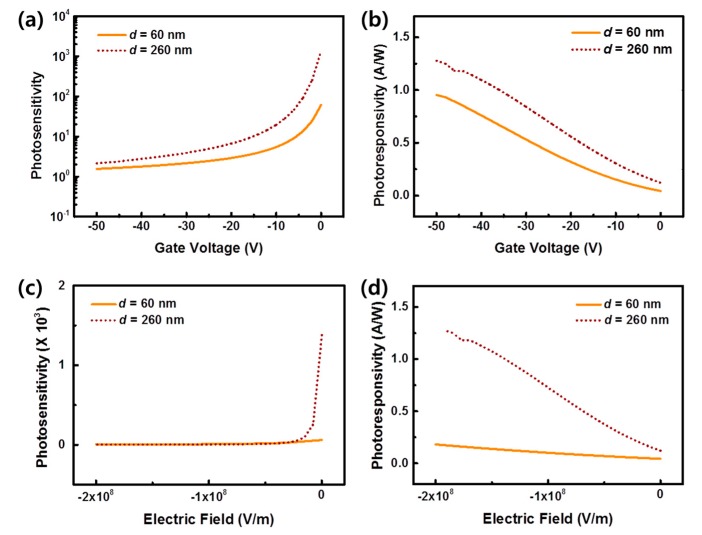
The photosensitivity and photoresponsivity of OPTs with PVP layers thickness *d* = 60 nm and *d* = 260 nm: (**a**) Photosensitivity and (**b**) photoresponsivity with various gate voltages; (**c**) Photosensitivity and (**d**) photoresponsivity under various electric fields.

In OPTs, the capacitance of the gate insulator (based on its thickness) is related to the charge accumulation capability and the resultant photoresponse of the device. That is, hydroxyl groups of the PVP gate insulator trap photogenerated electrons at the interface between organic semiconductor and PVP gate insulator in OPTs. Thus, the trapped electrons act as an additional negative gate voltage, increasing the drain current levels in p-type OPTs. Therefore, a higher accumulation of charges at the gate insulator (PVP)/pentacene interface could be preferred to obtain a high photoresponse of OPTs [14,16,18,19]. However, even if the OPT with the thinner PVP (*d* = 60 nm) had a higher capacitance and higher charge accumulation capability, it exhibited a Δ*V*_on_ lower than that of the device with the thicker gate insulator (*d* = 260 nm). Next, we evaluated the photosensitivity (*P*) and photoresponsivity (*R*) of the devices from Figure 2. As shown in Figure 3a,b in the electrically on state range (from *V*_g_ −50 to 0 V), both parameters were higher in the OPT with the thicker PVP layer compared with the thinner PVP. Specifically, the maximum values of *P* and *R* were 6.14 × 10^1^ and 0.95 A/W for *d* = 60 nm, respectively, and 1.38 × 10^3^ and 1.28 A/W for *d* = 260 nm, respectively. To compare the photoresponse properties between these OPTs with two different thicknesses of the gate insulators directly, we calculated *P* and *R* with respect to the electric field, as shown in Figure 3c,d, respectively. These graphs show the same tendency as those shown in Figure 3a,b. This indicates that the photocurrent by the photoinduced charge carriers was larger in the device with the thicker PVP of *d* = 260 nm than the photocurrent with the thinner PVP. Hence, we can conclude that another factor also affects the photoresponsive characteristics of the OPT other than the capacitance of the gate insulator and the resultant charge accumulation capability.

**Figure 4:**
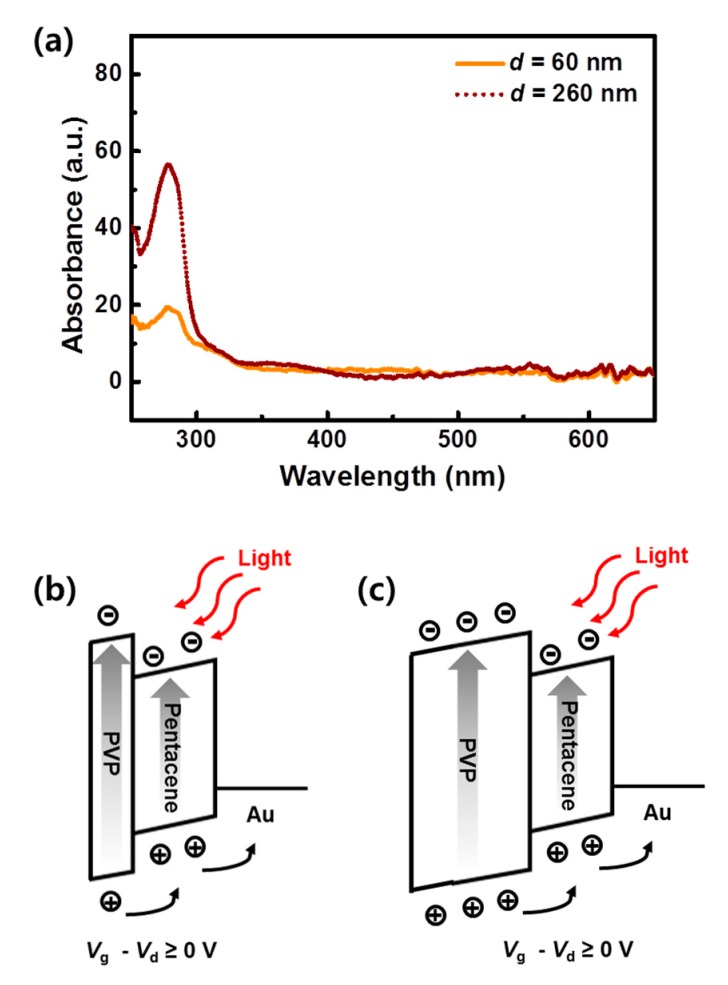
(**a**) UV-vis absorbance of PVP layers with thickness *d* = 60 and *d* = 260 nm. Schematics representing the operating mechanism of the OPTs with (**b**) *d* = 60 and (**c**) 260 nm.

Next, we discuss the origin of the high photoresponse of the OPT with the thicker PVP layer. As shown in Figure 4a, the absorbance of the PVP layers (*d* = 60 nm and 260 nm) in the UV-vis range was measured for investigating the photoresponsive properties of each film. It is noteworthy that PVP is known as a light-sensitized material. Therefore, the sole photoresponsive properties of the PVP gate insulators must be considered for analyzing the photoresponse of the PVP-based OPTs. As shown in Figure 4a, the thicker PVP layer absorbed significantly more UV light than the thinner layer. Based on this result, the higher photoresponse of the OPTs with the thicker PVP layer (*d* = 260 nm), despite the lower charge-accumulation properties, was discovered to be primarily attributed to the higher absorbance of photons by the PVP layer. Figure 4b,c show schematics representing the operating mechanisms of our devices. Owing to the electric field between the gate and drain electrodes (*V*_g_ - *V*_d_ ≥ 0), photogenerated holes were moved to the pentacene and then the Au (drain) electrodes thereby contributing to the photocurrent; in contrast, photogenerated electrons were trapped at the hydroxyl groups of the PVP layers. These trapped electrons acted as additional negative gate bias; therefore, optical memory characteristics were obtained [18,19,25]. Specifically, for the thinner PVP device (*d* = 60 nm) shown in Figure 4b, fewer electron–hole pairs were generated, leading to a lower photoresponse being obtained. Meanwhile, for the thicker PVP device (*d* = 260 nm) shown in Figure 4c, more electron–hole pairs were generated; therefore, a higher photoresponse was displayed by this thicker PVP-based device.

## 4. Conclusions

We herein presented the effect of the thickness of photoresponsive gate insulators (PVP) on the performance of OPTs. It was discovered that, between OPTs with two different thicknesses of PVP (*d* = 60 and 260 nm), the thicker PVP-based device showed a higher photoresponse than the thinner PVP device, i.e., the onset voltage shifted under light illumination, photosensitivity, and photoresponsivity. For the device with the thicker PVP layer, even though the accumulation capability of photogenerated charges was lower than that with the thinner PVP layer, more photons were absorbed in the photoresponsive gate insulator, resulting in a higher generation rate of electron–hole pairs under light exposure. That is, in our devices, the degree of light absorption by the photoresponsive PVP gate insulators primarily governed the photoresponse. Our physical description regarding the bulk effect of the photoresponsive insulators on the performance of the OPTs can provide a useful guideline for the design and construction of high-performance organic-based photosensing devices and systems.

## Supplementary Materials

The following are available online at https://www.mdpi.com/1996-1944/13/7/1565/s1, Figure S1. Real thickness data of the produced PVP layers (*d* = 60 and 260 nm). Actual thickness values indicated as *d* = 60 nm and 260 nm were about 55 nm and 255 nm, respectively. Figure S2. Comparison of gate leakage current of the OPTs with two different thicknesses of PVP (*d* = 60 and 260 nm) in the dark. Figure S3. Transfer curves of the OPTs with two different thicknesses, *d*, of PVP, in the dark and under different intensity light exposure (3 and 12 mW/cm^2^); (a) *d* = 60 nm and (b) *d* = 260 nm.
