# Supplementary material for: Improvement of Photoresponse in Organic Phototransistors through Bulk Effect of Photoresponsive Gate Insulators
Source: Materials (Basel). 2020 Mar 28;13(7):1565. doi: 10.3390/ma13071565 (PMC7178102; doi:10.3390/ma13071565)
Supplement: Supplementary file 1 [file materials-13-01565-s001.pdf]

materials

Supplementary Materials: Improvement of photoresponse in organic phototransistors through bulk effect of photoresponsive gate insulators

Hea-Lim Park [1], Min-Hoi Kim [2] and Hyeok Kim [3,*]

[1] Department of Materials Science and Engineering, Seoul National University, Kwanak-gu, Seoul 151-600, Republic of Korea; haelim1017@snu.ac.kr
[2] Department of Creative Convergence Engineering, Hanbat National University, Yuseong-ku, Daejeon 305-719, Republic of Korea; mhkim8@hanbat.ac.kr
[3] School of Electrical and Computer Engineering, University of Seoul, 163 Seoulsiripdaero, Dongdaemun-gu, Seoul 02504, Republic of Korea
* Correspondence: hyeok.kim@uos.ac.kr; Tel.: +82-2-6490-2354

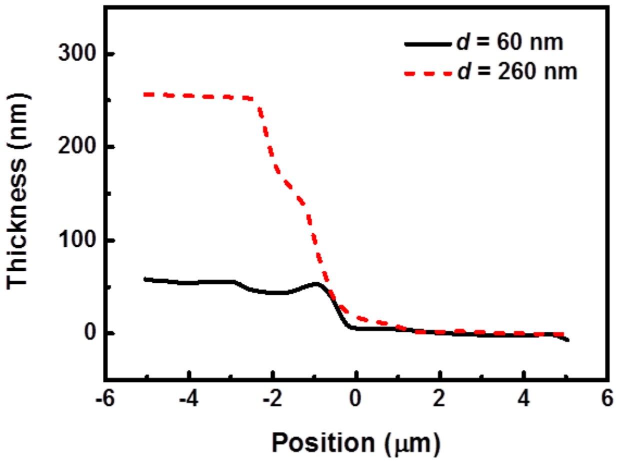

Figure S1. Real thickness data of the produced PVP layers (*d* = 60 and 260 nm). Actual thickness values indicated as *d* = 60 nm and 260 nm were about 55 nm and 255 nm, respectively.

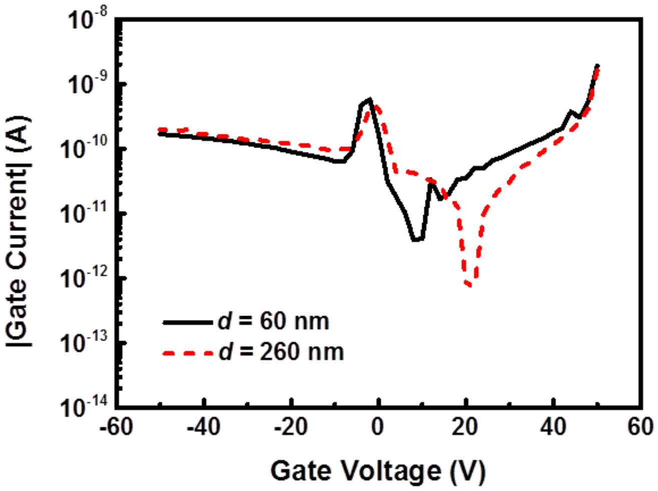

Figure S2. Comparison of gate leakage current of the OPTs with two different thicknesses of PVP (*d* = 60 and 260 nm) in the dark.

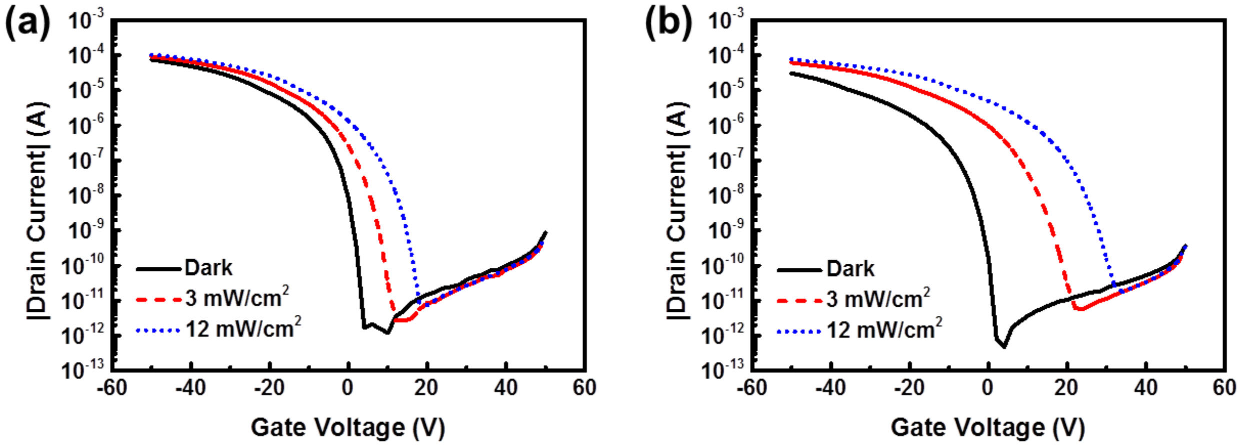

Figure S3. Transfer curves of the OPTs with two different thicknesses, *d*, of PVP, in the dark and under different intensity light exposure (3 and 12 mW/cm^2); (a) *d* = 60 nm and (b) *d* = 260 nm.

The onset voltage shift (ΔV_{on}) of the devices after light exposure was examined. It is noteworthy that the ΔV_{on} values have been widely investigated as a parameter for evaluating the photoresponse of OPTs. As shown in Fig. S3, under light illumination (3 mW/cm^2), the ΔV_{on} values of the *d* = 60 and 260 nm cases were 7 and 20 V, respectively. In addition, under higher light intensity of 12 mW/cm^2, the thicker device also showed same tendency, that is, higher ΔV_{on} value of 31 V than the thinner device (ΔV_{on} = 14 V). It should be noted that, as light intensity increased, ΔV_{on} values were enhanced.

© 2020 by the authors. Licensee MDPI, Basel, Switzerland. This article is an open access article distributed under the terms and conditions of the Creative Commons Attribution (CC BY) license (http://creativecommons.org/licenses/by/4.0/).
